# Rediscovery and lectotypification of *Diplazium velutinum* Holttum (Athyriaceae), a rare endemic fern from Peninsular Malaysia

**DOI:** 10.3897/phytokeys.276.197738

**Published:** 2026-06-11

**Authors:** Nur Aliah, Nurul Nadhirah, Haja Maideen, Nik Norhazrina, Shamsul Khamis, Syazwani Mohd Yusop, Syazwani Basir, Nur Syazwana, Danial Hariz Zainal Abidin, Nur Farah Ain Zainee

**Affiliations:** 1 Department of Biological Sciences and Biotechnology, Faculty of Science and Technology, Universiti Kebangsaan Malaysia, 43600 Bangi, Selangor, Malaysia Faculty of Science and Technology, Universiti Kebangsaan Malaysia Bangi Malaysia; 2 Glami Lemi Biotechnology Research Centre, Universiti Malaya, 71650 Kuala Klawang, Negeri Sembilan, Malaysia China-ASEA College of Marine Sciences, Xiamen University Malaysia Sepang Malaysia; 3 China-ASEA College of Marine Sciences, Xiamen University Malaysia, 43900 Sepang, Selangor, Malaysia Faculty of Tropical Forestry, Universiti Malaysia Sabah Kota Kinabalu Malaysia; 4 Faculty of Tropical Forestry, Universiti Malaysia Sabah, 88400 Kota Kinabalu, Sabah, Malaysia Glami Lemi Biotechnology Research Centre, Universiti Malaya Kuala Klawang Malaysia

**Keywords:** Athyriaceae, Cameron Highlands, endemic, montane forest, rediscovery

## Abstract

*Diplazium velutinum* Holttum (Athyriaceae) is a rare endemic fern species originally described in 1937 from Cameron Highlands, Pahang, Peninsular Malaysia. Since its original collection by R.E. Holttum, this species has remained poorly documented, with only a single subsequent record in 1988. During floristic surveys conducted in 2018, this species was rediscovered, representing the first documented collection in more than 30 years. A detailed morphological description is provided, the spore surface ornamentation is characterized, the molecular phylogenetic placement of the species within Athyriaceae is summarized, and ecological characteristics of its habitat are documented. The species is distinguished from its closest congener, *D. tomentosum*, by lamina dissection and stipe scale characters. *Diplazium velutinum* clusters together with *D. crenatoserratum* and *D. tomentosum*, consistent with its status as a distinct species. A lectotype is formally designated here for *D. velutinum* based on the original syntype S.F.N. 31221 held at the Singapore Botanic Gardens Herbarium (SING). This rediscovery demonstrates the value of continued pteridological surveys in the montane forests of Peninsular Malaysia and highlights the scientific significance of protecting montane forest habitats.

## Introduction

The fern genus *Diplazium* Swartz is the most species-rich genus in the family Athyriaceae, comprising approximately 300 species worldwide, with centers of diversity in tropical and subtropical regions (Wei et al. 2013; PPG I 2016; Hori and Kanemitsu 2021). Molecular phylogenetic studies have robustly supported *Diplazium* as a monophyletic genus characterized by linear sori, continuous grooves on the rachis and costa, and distinctive stipe scale morphology (Wang et al. 2003; Wei and Zhang 2020; Wei et al. 2025). In Peninsular Malaysia, 29 species of *Diplazium* have been recorded, of which six are endemic to the region: *D. angustipinna* Holttum, *D. insigne* Holttum, *D. kunstleri* Holttum, *D. procumbens* Holttum, *D. velutinum* Holttum, and an undescribed species (Parris and Latiff 1997; Maideen et al. 2019; Aliah et al. 2024, 2025).

*Diplazium velutinum* was described by Holttum (1937) based on specimens recorded in his series S.F.N. (= Singapore Field Number, the specimen numbering series used by Holttum for collections made during his tenure at the Singapore Botanic Gardens). One specimen was collected from Cameron Highlands, Pahang, in 1936 (S.F.N. 31221). Holttum (1937) also noted that *D. velutinum* is morphologically allied to *D. tomentosum* Blume, a common species of lowland forests, but distinguished the two by lamina dissection and scale characters.

Cameron Highlands, located in the Main Range of Peninsular Malaysia, encompasses the largest remaining area of pristine montane rainforest in the country. The district covers an area of 71,227 ha, with approximately 74% of the land situated above 1,000 m elevation, with average temperatures between 17–20 °C year-round, and receives annual rainfall exceeding 2,500 mm, creating optimal conditions for pteridophyte diversity (Mahamud et al. 2022). Holttum (1947) emphasized the high fern diversity of Cameron Highlands, and recent studies have continued to document significant finds from this area, including the first record of the genus *Cornopteris* for Peninsular Malaysia (Maideen et al. 2021) and new lycophyte species from the same montane zone (Kiew and Kamin 2018). However, the montane forests of Cameron Highlands have experienced significant habitat loss through agricultural encroachment and tourism development (Mahamud et al. 2022), underscoring the importance of continued floristic documentation. Here, the rediscovery of *D. velutinum* from the Mount Berinchang trail during floristic surveys in 2018 is reported. An updated morphological description and morphological key, first-record spore ornamentation data, a summary of its molecular phylogenetic placement, detailed ecological observations, and a formal lectotype designation resolving the nomenclatural status of the original type material are provided.

## Materials and methods

### Study area

**Figure 1.**
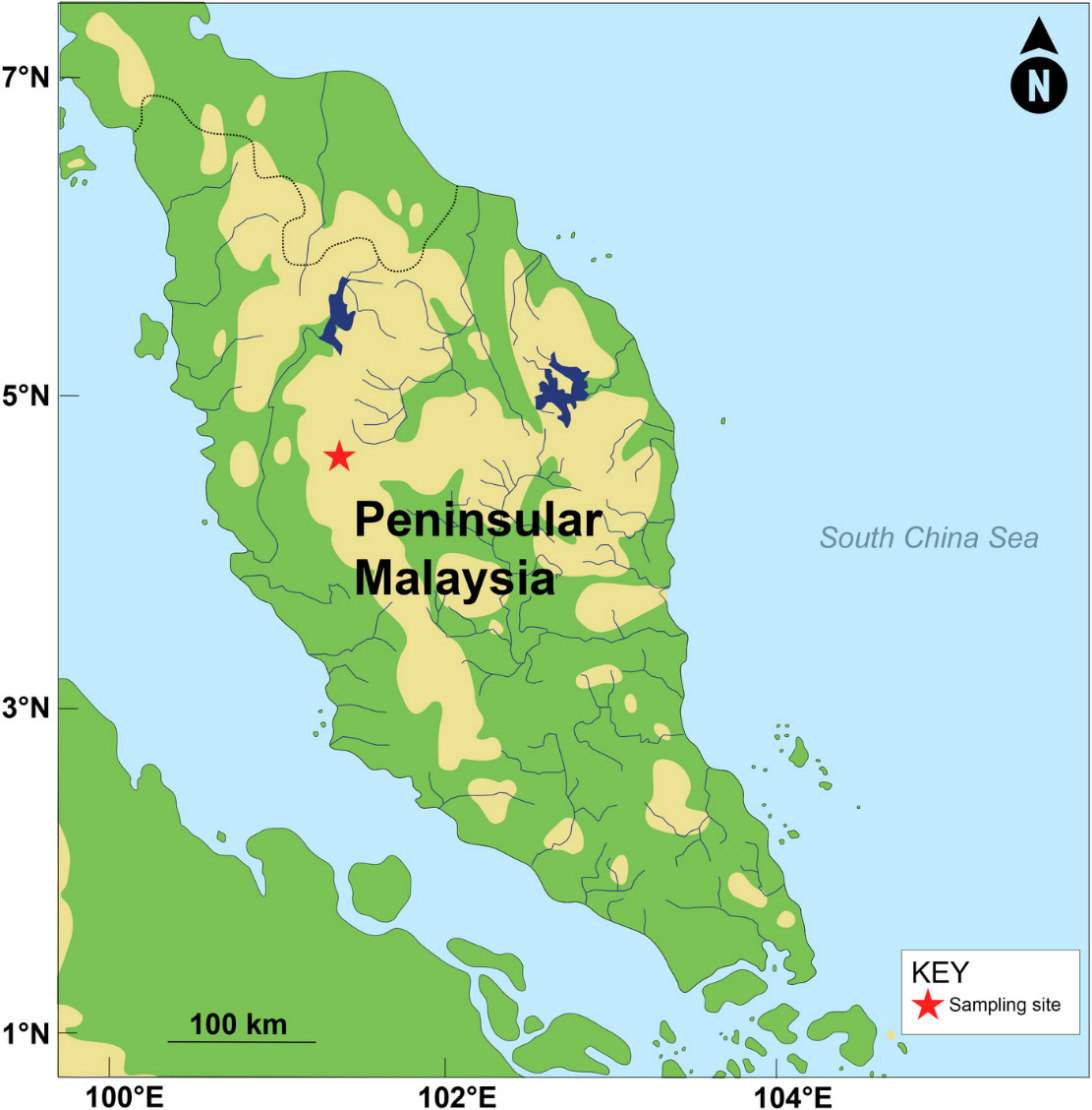
Map of Peninsular Malaysia showing the location of the collection site at the Mount Berinchang trail, Cameron Highlands, Pahang (red star).

Field collections were conducted along the trail to Mount Berinchang (4°31'N, 101°23'E), Cameron Highlands, Pahang, Peninsular Malaysia (Fig. 1). Mount Berinchang is the second-highest peak in Peninsular Malaysia at 2,031 m elevation. The study site is characterized by upper montane forest with a closed canopy dominated by oaks (Fagaceae), laurels (Lauraceae), and Myrtaceae. The forest floor is covered with dense mosses, liverworts, and ferns. Annual rainfall in the region exceeds 2,500 mm, with no distinct dry season, and mean annual temperature ranges from 17–20 °C.

### Specimen collection and morphological examination

**Figure 2.**
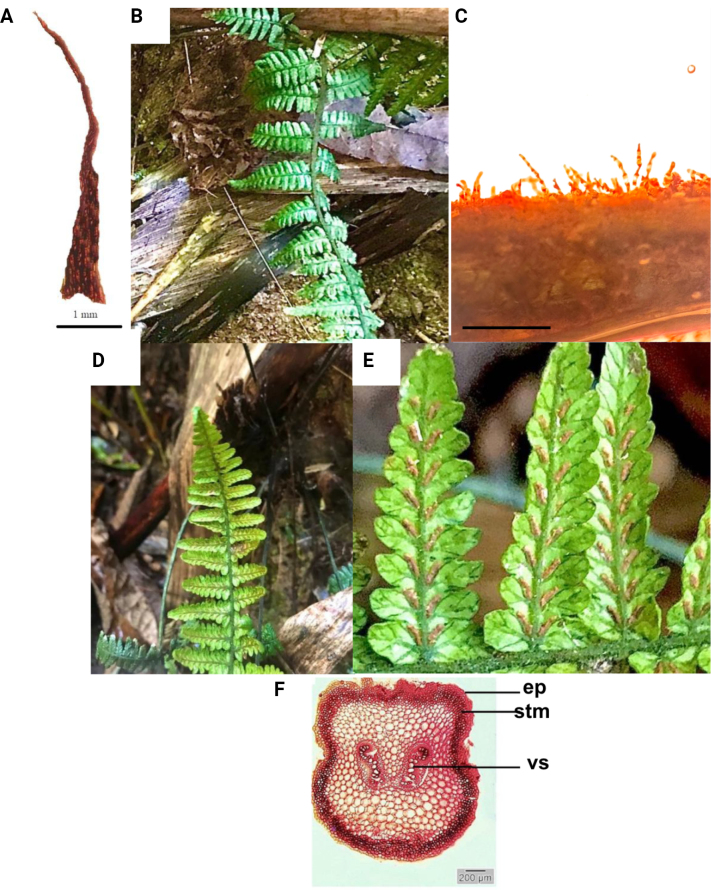
*Diplazium velutinum* Holttum. **A**. Stipe scale showing tapering, nearly black form; **B**. Habit in natural habitat showing bipinnatifid–tripinnatifid frond; **C**. Magnified view of the stipe base showing dense dark scales and multicellular hairs; **D**. Habit showing full frond architecture; **E**. Adaxial view of pinnae showing lobed pinnules; **F**. Transverse section of upper stipe showing epidermis (ep), sterome (stm), and U-shaped vascular strand (vs). Red-stained tissues represent lignified supporting tissues. Scale bars: 1 mm (**A, C**); 200 µm (**F**). Photographed by Nur Aliah.

Specimens of *D. velutinum* were collected in October 2018 from the trail to Mount Berinchang at an elevation of 1,687 m (Fig. 2). Voucher specimens were prepared following standard herbarium techniques and deposited at the Herbarium of Universiti Kebangsaan Malaysia (UKMB; acronym follows Thiers 2026, continuously updated). Field surveys were conducted specifically along the Mount Berinchang trail, based on historical collection records and recent documentation of high fern diversity from this locality. For morphological comparisons, herbarium images from the Royal Botanic Gardens, Kew (K), and the Singapore Botanic Gardens (SING) were consulted online. Physical specimens of the closely related *D. tomentosum*, *D. crenatoserratum*, and *D. prescottianum* from UKMB were examined for comparison. Morphological characters, including rhizome type, stipe and scale characteristics, lamina dissection, venation pattern, sori arrangement, and indusium morphology, were examined using a Meiji EMZ-5TRD stereo microscope. Ecological observations, including habitat type, elevation, associated species, and microhabitat preferences, were recorded at the collection site.

### Spore ornamentation

Mature spores from the specimens were prepared for field emission scanning electron microscopy (FESEM). Spores were mounted without chemical treatment onto aluminum stubs with adhesive carbon tape and coated with iridium via vacuum sputter coating. Observations were performed using a Thermo Fisher Quattro S field emission scanning electron microscope at the Electron Microscopy Unit, Faculty of Science and Technology, Universiti Kebangsaan Malaysia. Morphological terminology for perispore structures follows Tryon and Lugardon (1991), with modifications from Lellinger and Taylor (1997).

### Molecular phylogenetic framework

No new molecular data were generated for this study. Molecular phylogenetic placement of *D. velutinum* was derived from Aliah et al. (2024, 2025), which reconstructed the phylogeny of Athyriaceae in Peninsular Malaysia using five DNA markers (plastid: *rbcL*, *atpB*, *atpA*, *trnL-F*; nuclear: ITS) and both maximum likelihood (ML) and Bayesian inference (BI) methods. The topology from that analysis is used here to contextualize the taxonomic position of *D. velutinum*.

## Results

### Taxonomic treatment

#### Diplazium velutinum

Taxon classificationPlantaePolypodialesAthyriaceae

Holttum, Gardens’ Bulletin Straits Settlements 9: 126. 1937.

5081F89D-1CBA-5627-90AF-E373CCB9E305

##### Lectotype (designated here).

• R.E. Holttum S.F.N. 31221 (SING!); K [K000492397, K000451191, K000492398]! and L [L.3514169]!.

##### Syntypes.

• R.E. Holttum S.F.N. 31221 (SING!, K [K000492397, K000451191, K000492398]!, L [L.3514169]!), Pahang, Cameron Highlands, Jalan Telom, 47^th^ mile, 4°28'N, 101°23'E, 1,463 m elev., Malaysia, 14 May 1936; • R.E. Holttum S.F.N. 23428 (K!), Pahang, Cameron Highlands, 4,800 ft., MALAYSIA, 5 Apr 1930.

In his original treatment, Holttum listed two specimens under a single “Type” heading—S.F.N. 31221 and S.F.N. 23428—without designating a holotype and cited no additional material under a separate “Other specimens” entry, as he did for other species in the same paper. These two specimens therefore constitute syntypes, and lectotypification is required to fix the nomenclatural type.

S.F.N. 31221 is selected as the lectotype over S.F.N. 23428 for the following reasons. First, S.F.N. 31221 is the more complete and diagnostically informative specimen, collected from the same locality (Cameron Highlands, 47^th^ mile, Telom Road, 4,800 ft) explicitly cited in Holttum’s (1937) morphological description and ecological note that the species grows in “the wettest and shadiest mountain forest, near streams.” Second, S.F.N. 31221 is represented by five sheets across three herbaria—three sheets at Kew (K000492397, K000451191, K000492398), one at SING, and one at Naturalis (L)—providing exceptional accessibility for future workers, further supporting its selection over S.F.N. 23428, which is held at a single institution. Third, S.F.N. 23428, collected earlier in 1930 from a less precisely localized Cameron Highlands site, appears to have been cited by Holttum as a secondary supporting record. Designation of S.F.N. 31221 therefore best serves nomenclatural stability and is consistent with the intent of Art. 9.12 of the International Code of Nomenclature for algae, fungi, and plants (Turland et al. 2018), which recommends selecting the lectotype that most completely represents the protologue.

##### Description.

***Rhizome*** short, erect. ***Stipe*** to 40 cm long, densely clothed with multicellular brown hairs; ***scales*** nearly black, tapering, entire, to 10 × 0.8 mm. ***Lamina*** bipinnatifid–tripinnatifid, to 25 × 15 cm; rachis and costa with dense brown indument. ***Pinnae*** 12–15 pairs; sub-basal pinnae largest, to 8 × 3 cm. ***Pinnules*** to 12 × 4 mm, margin ciliate or lobed to halfway to costule. ***Venation*** free, 7–10–jugate. ***Sori*** diplazioid; indusium thin, broad. ***Stele*** in transverse section at upper stipe.

##### Differential diagnosis.

*Diplazium velutinum* differs from *D. tomentosum* (Table 1) in its bipinnatifid–tripinnatifid lamina (vs. pinnate–bipinnatifid), nearly black stipe scales to 10 mm long (vs. pale brown, ca. 4 mm), indument covering the entire stipe and abaxial lamina (vs. restricted to costa and rachis), and restriction to upper montane forest above 1,400 m.

**Table 1.** Morphological comparison of *Diplazium velutinum* and three allied species.

Character	D. velutinum*	*D. tomentosum*	*D. crenatoserratum*	*D. prescottianum*
Frond size (cm)	To 25 × 15	to 35 × 15 (usually smaller)	To 30 × 12	To 20 × 10
Stipe length (cm)	To ~40	To ~40	To ~35	To ~30
Rhizome	Short, erect	Short, erect	Short, erect	Short, erect
Stipe scale size (mm)	10 × 0.8	4 × 0.5	3–4 × 0.5	< 6 × 0.4
Scale margin	Entire	Entire	Dentate	Dentate
Scale color	Blackish brown	Reddish brown	Dark brown	Reddish brown
Lamina dissection	Bipinnatifid–tripinnatifid; pinnate at pinna base only, otherwise lobed to costa	Pinnate–bipinnatifid	Bipinnatifid	Bipinnate
Pinna base	Truncate, tapering to acute–acuminate apex; pinnules at 90° to rachis	Cuneate; upper pinnae often auriculate	Cuneate	Cuneate
Rachis indument	Stellate hairs	Stellate hairs	Stellate hairs (dense)	Sparse hairs
Spore ornamentation	Cristate with alate folds and irregular gaps between adjacent folds	Broadly winged perispore folds	Interconnected folds forming a mesh-like pattern	Low rugate folds
Stele shape (upper stipe)	U–shaped	U–shaped	U–shaped	U–shaped
Pinnule margin	Ciliate or lobed to 1/2 costule	Lobed to 1/3–1/2 costule	Serrate–dentate	Serrate
Elevation range	1,463–1,687 m	Lowland to 800 m	200–900 m	400–1,200 m

* Data based on fresh material and UKMB specimens collected in 2018 (NA2018-49, NA2018-52).

### Key to *Diplazium velutinum* and *D. tomentosum*

**Table:** 

1	Lamina pinnate; scales pale brown; indument restricted to abaxial costa and rachis; lowland to lower montane forest	***D. tomentosum***
–	Lamina bipinnatifid–tripinnatifid; scales nearly black; indument covering entire stipe and abaxial lamina; upper montane forest	***D. velutinum***

### Spore ornamentation

**Figure 3.**
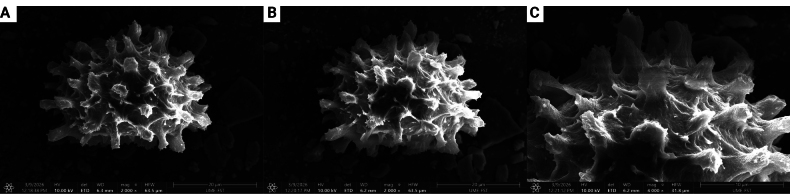
Spore ornamentation of *Diplazium velutinum* from field emission scanning electron microscopy (Thermo Fisher Quattro S FEG-SEM, 10.00 kV, ETD detector). **A**. Whole spore in distal polar view (2,000×) showing broadly winged-alate perispore folds radiating from the spore body, producing a conspicuously stellate to lobate outline; irregular gaps visible between adjacent perispore folds; fold margins strongly lacerate and irregularly dissected; **B**. Whole spore in oblique lateral view (2,000×) confirming the genuinely free-standing, projecting nature of the perispore folds; fenestrations most prominent toward the mid-region, with the finely granular spore body surface visible through the gaps; **C**. Close-up of upper perispore surface (4,000×) showing broad membranous folds with strongly lacerate margins, some subdivided into narrow finger-like projections; fold faces with secondary micro-rugulate texture and sparse globular surface deposits; fold bases loosely anastomosing. Scale bars: 20 µm (**A**); 20 µm (**B**); 10 µm (**C**).

All spores of *D. velutinum* are monolete and bilaterally symmetrical, consistent with the condition reported for other *Diplazium* species from Peninsular Malaysia (Aliah et al. 2025). The perispore ornamentation of *D. velutinum* is characterized by broadly winged-alate folds with irregular gaps between adjacent folds (Fig. 3). At lower magnification, spores viewed from the distal face display a prominently sculptured outline formed by large, thin, sheet-like folds radiating from the spore body, giving the spore a conspicuously stellate to lobate outline in polar view. The folds are confirmed as genuinely free-standing, projecting structures that extend well above the spore body surface rather than representing surface relief features, as clearly resolved from the oblique lateral view. Irregular gaps are present between adjacent folds, most prominent toward the mid-region of the spore surface, through which the finely granular spore body surface is visible. Individual folds are broad and membranous, with free margins that are strongly lacerate and irregularly dissected; some margins are further subdivided into narrow, finger-like projections at their distal tips. The faces of the folds exhibit a secondary micro-rugulate texture, distinct from the primary fold architecture, with sparse globular to subglobular surface deposits visible on the fold surfaces. Fold bases loosely anastomose, forming an interconnected arrangement across the basal perispore surface. No discrete granular or echinate elements are present independently of the micro-rugulate fold texture.

This combination of broadly winged-alate folds, lacerate fold margins, and micro-rugulate fold surfaces places the perispore ornamentation of *D. velutinum* within the wing-like projection type *sensu*Lellinger and Taylor (1997). Comparative examination of previously published FESEM images of *D. tomentosum* and *D. crenatoserratum* (Aliah et al. 2025) indicates that *D. velutinum* is intermediate between the broadly developed wing-like perispore folds of *D. tomentosum* and the interconnected mesh-like fold pattern observed in *D. crenatoserratum*. However, *D. velutinum* is distinguished by its conspicuously stellate to lobate outline in polar view, produced by the radiating arrangement of broadly winged folds. These differences in perispore architecture are consistent with the phylogenetic position of *D. velutinum* as sister to both species in Subclade IE (Aliah et al. 2024).

### Ecology and habitat

*Diplazium velutinum* occurs in upper montane forest at elevations from 1,463 to 1,687 m. At the collection site on the Mount Berinchang trail, the species was found growing terrestrially on steep, well-drained slopes in deep shade beneath a closed forest canopy. Associated pteridophytes observed in the surrounding habitat included *Athyrium anisopterum*, *Cornopteris opaca*, and various filmy ferns (Hymenophyllaceae). The population of *D. velutinum* was also observed growing beneath wild banana plants (*Musa* sp.) along moist, shaded slopes within upper montane forest. The habitat was characterized by dense bryophyte cover, high humidity, and deep accumulations of humus-rich leaf litter beneath a closed canopy. The substrate consisted of humus-rich soil with a thick layer of decomposing leaf litter. Air temperature at the collection site ranged from 15–18 °C, and the relative humidity exceeded 90%. The species appears to be highly restricted to undisturbed montane forest with continuous canopy cover and high atmospheric moisture. Holttum (1937) noted that *D. velutinum* represents the montane derivative of the lowland *D. tomentosum*, suggesting ecological replacement along an elevational gradient. These observations support this hypothesis, as *D. tomentosum* is typically found at lower elevations (below 800 m) in hill dipterocarp and lower montane forests, while *D. velutinum* appears restricted to upper montane zones above 1,400 m. This pattern of elevational partitioning is common among closely related fern species in tropical mountains and is consistent with the ecological differentiation hypothesis for their origin.

### Specimens examined

R.E. Holttum S.F.N. 31221 (SING [lectotype], K [K000492397, K000451191, K000492398] [isolectotypes], L [L.3514169] [isolectotype]), Pahang, Cameron Highlands, Jalan Telom, 47^th^ mile, 4°28'N, 101°23'E, 1,463 m elev., Malaysia, 14 May 1936. R.E. Holttum S.F.N. 23428 (K!), Pahang, Cameron Highlands, 4,800 ft., Malaysia, 5 Apr 1930. Nur Aliah & Nurul Nadhirah NA2018-49 (UKMB), Pahang, Cameron Highlands, trail to Mount Berinchang, 4°31'N, 101°23'E, 1,687 m elev., Malaysia, 5 Oct 2018. Nur Aliah & Nurul Nadhirah NA2018-52 (UKMB), Pahang, Cameron Highlands, trail to Mount Berinchang, 4°31'N, 101°23'E, 1,687 m elev., Malaysia, 5 Oct 2018.

## Discussion

The rediscovery of *Diplazium velutinum* more than 30 years after Piggott’s record (Piggott 1988) confirms the continued presence of this rare endemic species in the montane forests of Cameron Highlands. The additional characters documented here—including scale dimensions, abaxial lamina indument, spore micromorphology, and ecological associates—collectively reinforce the morphological distinctiveness of *D. velutinum* and substantially expand the diagnostic information available beyond Holttum’s (1937) original protologue description.

### Morphological distinctness and relationships

Holttum’s (1937) suggestion that *D. velutinum* represents a montane derivative of *D. tomentosum* is consistent with the observations reported here. The two species share erect rhizomes, stellate hairs on the rachis and costae, and similar sorus morphology. However, *D. velutinum* is clearly distinguished by its larger fronds, longer blackish-brown scales (to 10 × 0.8 mm) with entire margins, and more highly dissected bipinnatifid–tripinnatifid lamina. These morphological differences are accompanied by marked ecological differentiation: *D. velutinum* is restricted to upper montane forest above 1,400 m, while *D. tomentosum* occurs predominantly in lowland to lower montane forests below 800 m. The identification key provided here makes these differences explicit and facilitates reliable field identification of all four morphologically similar species in the region. The combination of scale length, scale color, lamina dissection, and elevation together provides unambiguous separation. The U-shaped stele observed in the upper stipe of *D. velutinum* is consistent with the condition reported across multiple athyrioid fern genera in Peninsular Malaysia and has been recognized as a useful supplementary anatomical character in regional fern systematics (Maideen et al. 2023).

### Molecular phylogenetic placement

Molecular phylogenetic analyses of Athyriaceae in Peninsular Malaysia (Aliah et al. 2024; voucher NA2018-49, UKMB) place *D. velutinum* in a well-supported subclade (Subclade IE, 94% bootstrap support) together with *D. crenatoserratum* and *D. tomentosum*. This grouping is corroborated by the global sectional revision of *Diplazium* by Wei et al. (2025), which places these species in section *Anisogonium* based on combined plastid genomic, nuclear ribosomal, and morphological evidence, a placement consistent with the recent addition of *D. clivicolum* to section *Anisogonium* from southern Yunnan, China (Wei and Zuo 2025). The molecular data unambiguously reject treating *D. velutinum* as a variety of *D. tomentosum*, as sister-group relationships are consistently resolved with good support across analyses. This molecular framework also provides evolutionary context for interpreting the morphological and ecological divergence between *D. velutinum* and *D. tomentosum*: the two species likely diverged through ecological speciation associated with altitudinal range partitioning along a montane gradient, a pattern broadly documented in tropical fern diversification (Wei et al. 2013). The absence of *D. velutinum* from the plastome phylogeny of Wei et al. (2025) reflects the fact that *D. velutinum* was known only from herbarium material at the time of that study, predating the present rediscovery; this gap in global sampling highlights the value of this rediscovery and the availability of fresh material for future molecular work.

### Spore ornamentation and systematic significance

The spore surface ornamentation of *D. velutinum*—characterized by broadly winged-alate folds with irregular gaps between adjacent folds and micro-rugulate fold surfaces—occupies a morphological position between the broadly developed wing-like perispore folds of *D. tomentosum* and the interconnected mesh-like fold pattern of *D. crenatoserratum* (Aliah et al. 2025). This pattern is congruent with the phylogenetic position of *D. velutinum* as sister to *D. crenatoserratum* and *D. tomentosum* in Subclade IE. Spore ornamentation in Athyriaceae has been highlighted as a reliable supplementary taxonomic character (Liu et al. 2000; Aliah et al. 2025), and the present data confirm its value for *D. velutinum*, providing additional evidence for its specific status. This is the first reported FESEM examination of *D. velutinum* spores, filling an important gap in regional pteridological knowledge. Comparative examination of previously published FESEM images of *D. tomentosum* and *D. crenatoserratum* (Aliah et al. 2025) further supports the distinctiveness of *D. velutinum* through its conspicuously stellate to lobate outline in polar view. Although additional comparative FESEM observations based on authenticated material would be valuable, the available evidence already demonstrates the taxonomic significance of spore ornamentation within this species complex.

## Supplementary Material

XML Treatment for Diplazium velutinum

## Additional information

### Artificial Intelligence (AI) use

The authors accept full responsibility for the content of the manuscript, including the disclosure of any use of AI.

No AI tools were used in the preparation of this manuscript.

### Author ORCIDs

N. Aliah https://orcid.org/0009-0002-9558-8849

N. Nadhirah https://orcid.org/0009-0008-6380-8767

N. Norhazrina https://orcid.org/0000-0001-5790-9811

S. Khamis https://orcid.org/0000-0002-8247-7990

S. Mohd Yusop https://orcid.org/0000-0001-6538-4138

D.H. Zainal Abidin https://orcid.org/0000-0002-2654-6306

### Data availability

All of the data that support the findings of this study are available in the main text.
